# Continuous and low-energy ^125^I seed irradiation changes DNA methyltransferases expression patterns and inhibits pancreatic cancer tumor growth

**DOI:** 10.1186/1756-9966-30-35

**Published:** 2011-04-02

**Authors:** Jian-xia Ma, Zhen-dong Jin, Pei-ren Si, Yan Liu, Zheng Lu, Hong-yu Wu, Xue Pan, Luo-wei Wang, Yan-fang Gong, Jun Gao, Li Zhao-shen

**Affiliations:** 1 Department of Gastroenterology, The Changhai Hospital, The Second Military Medical University, Shanghai, PR China

**Keywords:** ^125^I Seed Irradiation, Pancreatic Cancer, DNA methyltransferases, DNA hypomethylation, Apoptosis

## Abstract

**Background:**

Iodine 125 (^125^I) seed irradiation is an effective treatment for unresectable pancreatic cancers. However, the radiobiological mechanisms underlying brachytherapy remain unclear. Therefore, we investigated the influence of continuous and low-energy ^125^I irradiation on apoptosis, expression of DNA methyltransferases (DNMTs) and cell growth in pancreatic cancers.

**Materials and methods:**

For *in vitro *^125^I seed irradiation, SW-1990 cells were divided into three groups: control (0 Gy), 2 Gy, and 4 Gy. To create an animal model of pancreatic cancer, the SW 1990 cells were surgically implanted into the mouse pancreas. At 10 d post-implantation, the 30 mice with pancreatic cancer underwent ^125^I seed implantation and were separated into three groups: 0 Gy, 2 Gy, and 4 Gy group. At 48 or 72 h after irradiation, apoptosis was detected by flow cytometry; changes in DNMTs mRNA and protein expression were assessed by real-time PCR and western blotting analysis, respectively. At 28 d after ^125^I seed implantation, *in vivo *apoptosis was evaluated with TUNEL staining, while DNMTs protein expression was detected with immunohistochemical staining. The tumor volume was measured 0 and 28 d after ^125^I seed implantation.

**Results:**

^125^I seed irradiation induced significant apoptosis, especially at 4 Gy. DNMT1 and DNMT3b mRNA and protein expression were substantially higher in the 2 Gy group than in the control group. Conversely, the 4 Gy cell group exhibited significantly decreased DNMT3b mRNA and protein expression relative to the control group. There were substantially more TUNEL positive in the ^125^I seed implantation treatment group than in the control group, especially at 4 Gy. The 4 Gy seed implantation group showed weaker staining for DNMT1 and DNMT3b protein relative to the control group. Consequently, ^125^I seed implantation inhibited cancer growth and reduced cancer volume.

**Conclusion:**

^125^I seed implantation kills pancreatic cancer cells, especially at 4 Gy. ^125^I-induced apoptosis and changes in DNMT1 and DNMT3b expression suggest potential mechanisms underlying effective brachytherapy.

## Introduction

Pancreatic cancer is a devastating disease that is generally detected at a late stage. Surgical resection is the only potentially curative treatment; however, only 10 to 20% of patients are candidates for curative surgical resection due to advanced diagnosis, poor patient condition and tumor location. The remaining patients have to seek alternative therapies [1-3]. Even with resection, long term survival remains poor, with a median survival of 12 - 20 months. The survival rate of pancreatic cancer patients is so short, that treatment tends to be palliative. Recently, palliative surgery, endoscopic drainage, chemotherapy or brachytherapy alone or in combination have been used to elongate the survival and alleviate pain or jaundice symptoms [4-7].

Iodine-125 (^125^I) brachytherapy with either external beam radiation therapy (EBRT) or interstitial brachytherapy (IBT) improve local control and increase survival [8-10]. However, EBRT requires high doses of irradiation for efficacy [8]. Moreover, the very radioresponsive organs surrounding the pancreas adversely affect the dose of radiation used to target the tumor on radiation treatment [9]. Fractionated EBRT is only effective on cancer cells before metastasis occurs, and the efficiency of EBRT is usually impaired because, between irradiation treatments, tumor cells in the stationary phase enter the mitotic stage [8,9]. As a result, IBT has been introduced as treatment for unresectable pancreatic cancers to maximize local dose and minimize irradiation of the surrounding normal tissue [10]. Recently, ^125^I seed implantation, an efficient IBT technique, has attracted increasing attention because of its specific advantages: 1) effective irradiation dose applied in a single procedure; 2) reduced irradiation outside the target tumor; 3) elongating the tumor killing over several weeks or months; 4) percutaneous implantation under the guidance of ultrasound or CT [11,12].

Cancer irradiation therapy may keep tumor cells in the sensitive resting period, resulting in tumor cell apoptosis, inducing epigenetic changes to reactivate silenced tumor suppressor genes, and damaging DNA to kill the cancer cells. However, the radiobiological effect of persistent and low-energy ^125^I irradiation, especially on epigenetic modifications and apoptosis are not fully understood. Cancer cell apoptosis is an indicator of response to cancer treatment. Aberrant DNA methylation in cancer cells is a critical epigenetic process involved in regulating gene expression. DNA hypermethylation is associated with tumor suppressor gene silencing and defects in cell cycle regulation, resulting in tumor development and progression [13,14]. The DNA methyltransferases DNMT1, DNMT3a, and DNMT3b are the three main functional enzymes that are responsible for establishing and maintaining DNA methylation patterns in mammalian cells. The purpose of this study is to investigate the effect of persistent and low-energy ^125^I seed irradiation on apoptosis and the expression patterns of DNMTs in a mouse model of pancreatic cancer.

## Materials and methods

### Cell lines and cell culture

Human SW-1990 pancreatic cancer cell lines obtained from the American Type Culture Collection (Manassas, VA) were maintained in DMEM (pH 7.4; Sigma, St. Louis, MO) supplemented with 10% fetal bovine serum, 100 U/ml penicillin and 10 ng/ml streptomycin in a humidified atmosphere of 95% air and 5% CO_2 _at 37°C.

### *In vitro *^125^I seed irradiation model

**Figure 1:**
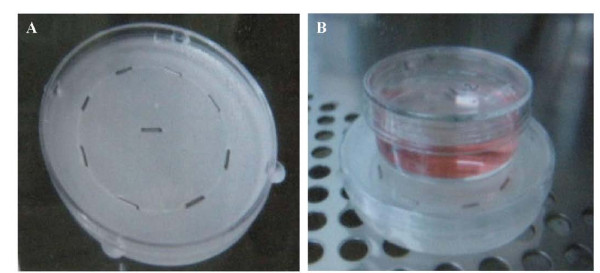
**^125^I seed irradiation model developed in-house**. In a 60-mm cell culture dish, eight ^125^I seeds were embedded in the solidified paraffin evenly around the circumference of a 35-mm diameter, and one ^125^I seed was placed at the center of dish. This arrangement produced a homogeneous dose distribution at the top of the cell culture dish, so that a 35-mm cell culture dish containing SW-1990 cells could be placed on it during the experiment.

Model 6711 ^125^I were kindly provided by Beijing Research Institute of Medical Science Lin Chung. A single seed is 0.84 mm in diameter, 4.5 mm long, has a surface activity of 22.2 MBq, a half-life of 60.2 d, and main transmission of 27.4 - 31.4 Kev X-ray and 35.5 Kev γ-ray. Liquid paraffin was poured into a 6-cm diameter cell culture dish. After the liquid solidified, there was a 5-mm height distance between the surface of the solid wax and the top of culture dish. In the paraffin plaque, eight ^125^I seeds were evenly embedded within recesses (4.5 mm × 0.8 mm) around a 35 mm diameter circumference, with one ^125^I seed placed in the center of the 60-mm dish (Figure 1A), in order to obtain a relatively homogeneous dose distribution at the top of the cell culture dish. A 35-mm culture dish was placed on the in-house ^125^I irradiation model during the experiment (Figure 1B). The culture dish was kept in the incubator to maintain constant cell culture conditions. The model was validated with thermoluminescent dosimetry measurement using an empirical formula from the American Association of Physicists in Medicine (AAPM; 15). The absorbed dose for different exposure time in various planes was also measured and verified. The exposure time for delivering doses of 2 Gy and 4 Gy are 44 and 92 h, respectively.

### ^125 ^I irradiation and Cell Group

The adherent SW-1990 cells were detached by 0.25% trypsin-EDTA until cells became a single cell suspension when observed under the microscope. The digestion was terminated by adding DMEM containing 10% fetal calf serum. The single cell suspension was diluted to a concentration of 1 × 10^5 ^cells/ml and was transferred to culture dishes with DMEM. Exponentially-growing SW1990 cells in a cell culture dish were irradiated using the in-house ^125^I seed irradiation model. The cell culture dishes were placed on the top of the *in vitro *^125^I seed irradiation model and placed in the incubator. The culture dishes were rotated clockwise at specific time intervals to guarantee even irradiation of the cells. The cultured cells were randomly divided into three groups: control group (0 Gy, without the embedded seed in the paraffin), 2 Gy, and 4 Gy.

### Apoptosis analysis by flow cytometry

Adherent SW 1990 cells cells were trypsinized and centrifuged for 5 min at 220x*g*. Cells were then washed three times in ice cold PBS and suspended in binding buffer (0.01 M Hepes, pH 7.4; 0.14 M NaCl; 2.5 mM CaCl_2_) at 1 × 10^6 ^cells/ml. The cells were stained with annexin V-FITC (1 μl/ml) and propidium iodide (5 μg/ml) for 15 min in the dark as described previously [15]. Cells were analyzed by fluorescence-activated cell sorting (FACS) using a Coulter EPICS and MOdFit SOFTWARE (Verity Software House, Topsham, MN). Each test was performed 3 times.

### Real-time polymerase chain reaction (PCR)

Total RNA was retracted from SW 1990 cells using Trizol reagent (Invitrogen, Carlsbad, CA). The housekeeping gene glyceraldehyde-3-phosphate dehydrogenase GAPDH was used as an internal reference [16]. Real-time PCR was performed by using the following primers: for DNMT1, upstream primer 5'-GTGGGGGACTGTGTCTCTGT-3' downstream primer 5'-TGAAAGCTGCATGTCCTCAC-3', and amplified fragment length of 204 bp; for DNMT3a, upstream primer 5'-ATCTCGCGATTTCTCGACTC- 3', downstream primer 5'-GCTGAACTTGGCTATCCTGC -3', and amplified fragment length of 180 bp; for DNMT3b, upstream primer 5'-TTGAATATGAAGCCCCCAAG- 3', downstream primer 5'-TGATATTCCCCTCGTGCTTC -3', amplified fragment length of 160 bp; for GAPDH, upstream primer 5'-GCACCGTCAAGGCTGAGAAC-3', downstream primer 5'-ATGGTGGTGAAGACGCCAGT-3', amplified fragment length of 142 bp. Cycling parameters: pre-denaturation 1 min, 95°C; denaturation 15 s, 95°C; annealing 15 s, 60°C; extension 45 s, 72°C, 40 cycles; final extension 5 min, 70°C. The PCR was repeated three times for each sample. The standard curve was generated with the ABI 7500 Real Time PCR system (Applied Biosystems, Carlsbad, CA, USA) to describe the linear relationship between threshold cycle (Ct) value and relative quantity (RQ). RQ values were obtained from measured Ct value with the following formula: 2^(-ΔΔCt)^, where ΔΔCt = ΔCt_T_; ΔCt_S _= (ΔCt_T _- ΔCt_TE _) - (ΔCt_S _- ΔCt_SE_), T is the target sample, S is the SW-1990 cell sample, and E is the reference. The RQ of mRNA in all groups were calculated relative to the RQ value in control group 1.

### Western blotting

Western blotting was performed as described previously [17,18]. Nuclear protein was prepared from SW-1990 pancreatic cancer cells with a Nuclear Protein extraction kit (Fermentas, Ontario, CA). The total protein concentration was determined by the Bradford assay using the Coomassie Protein Assay Reagent Kit (Pierce Biotechnology, Rockford, IL). Prepared protein samples (20 μg each) were boiled for 5 min and loaded onto a 12% SDS polyacrylamide gel. After separation by electrophoresis and electroblotting to nitrocellulose membranes, membranes were blocked by with 5% nonfat dry milk in 0.05% Tween 20 Tris-buffered saline (TTBS) at 4°C for 2 h. Membranes were incubated with rabbit anti-human anti-DNMT1 antibody (1:1000; Abcam, Cambridge, MA), DNMT3a (1:1000; Epitomics, Burlingame, CA) and DNMT3b (1:1000; Imagenex, Port Coquitlam, BC) at room temperature overnight. After three washes with TTBS, blots were incubated with horseradish peroxidase (HRP)-conjugated goat anti-rabbit IgG antibody (1:5000) for 2 h at room temperature. The membranes were visualized with an enhanced chemiluminescence (ECL) detection system (Pierce) and images acquired using a Fluores-max instrument (Alpha Innotech, Santa Clara, CA). The gray scale value of the respective bands was quantified using Quantity One imaging software (Bio-Rad Laboratories, Hercules, CA).

### Animal model of pancreatic cancer and animal group

The animals used in this study received humane care in compliance with the Guide to the Care and Use of Experimental Animals formulated by the Medical Ethical Committee on animal experiments of the Second Military Medical University. Twenty four 4 week old nude mice weighing 18 to 20 g were anesthetized by intraperitoneal injection of sodium pentobarbital (50 mg/kg). In a mini-laparotomy, the recipient rat pancreas was exposed and a small stab wound made in the pancreas parenchyma with a knife blade. The SW1990 cell suspension (1 × 10^5 ^cells/ml, 0.2 ml) was inoculated under the parenchyma of the pancreatic tail. Any leakage of the cell suspension into abdominal cavity was carefully removed with 75% ethanol to avoid peritoneal metastasis. Ten days later, the ultrasonic images demonstrated the formation of *in situ *pancreatic cancer with a tumor diameter of 1.52 ± 0.31 cm.

After the diagnosis of pancreatic cancer was established by ultrasound images during laparotomy, the 18-gauge needles were implanted into the visible mass at the tail of pancreas, and spaced in a parallel array at intervals of approximately 0.5 cm. After the needles were implanted, ^125^I seeds were implanted using a Mick-applicator with the spacing maintained at approximately 0.5 cm. The mice with pancreatic cancer were randomly divided into three groups. Groups I, II, and III underwent the implantation of 0 Gy, 2 Gy, and 4 Gy ^125^I seeds, respectively. The 2 Gy or 4Gy irradiation were achieved through implantation of 1 or 2 seeds, respectively, into the pancreatic tumor. The ^125^I seed have a average activity of 0.5 - 0.8 mCi. No seed implantation was performed in the 0 Gy irradiation group. After ^125^I seed implantation, two mice in the 0 Gy group died; however, no death was observed in the 2 Gy and 4 Gy groups.

### Measurement of tumor volume by ultrasonic images

Ultrasonic inspection was performed through using a GF-UCT240-AL5 (Olympus Co Ltd, Tokyo, Japan) endoscopic ultrasound (EUS) 0 and 28 d post-implantation with a probe frequency of 12 MHz. After anesthetizing the animals by intraperitoneal injection of sodium pentobarbital (50 mg/kg), the mouse abdomen was soaked with sterile deionized water. The ultrasonic images were acquired using the EUS probe with a water bag and the direct contact method. The long (a) and short (b) diameters were measured from the ultrasonic images. The volume of tumor was calculated according to the following formula: a × b^2^/2.

### TUNEL staining

TUNEL staining was described previously [19]. Formalin-fixed tissues were dehydrated, embedded in paraffin, and sectioned. Tissue sections were deparaffinized with xylene and rehydrated with graded dilution of ethanol and fixed by 4% paraformaldehyde. The tissue sections were incubated in 0.1% Triton X-100 in 0.1% sodium citrate (SSC) for 15 min and 0.3% H_2_O_2 _for 3 - 5 min. The slides were washed three times in phosphate-buffered saline (PBS) and incubated with 50 μl of TUNEL reaction mixture (TdT and fluorescein-labeled dUTP) in a humid atmosphere for 60 min at 37°C. After three washes in PBS, the sections were incubated for 30 min with an antibody specific for fluorescein-conjugated horseradish peroxidase. The TUNEL stain was visualized with a DAB substrate system in which nuclei with DNA fragmentation stained brown. Slides were mounted in neutral gum medium and were observed with an IX71 light microscope (Olympus, Tokyo, Japan). A commercial fluorometric TUNEL system (DeadEnd; Promega, Madison, WI) was used for analysis of apoptosis. Tissue sections were examined microscopically using a 40× objective; apoptotic cells were counted in 200 fields. Alternatively, lenses were dissected from Formalin-fixed eyeballs and pictures were taken with an MZ FLIII stereomicroscope (Leica Microsystems, Deerfield, IL) with bright-field transmitted light. All pictures were processed in ImageJ to measure the surface area and height of each lens for comparison.

### Immunohistochemical staining

Immunohistochemical analysis was conducted as described previously [20]. Tissues were obtained from pancreatic cancer approximately 5 mm distant from the center of the implanted ^125^I seed. Formalin-fixed tissues were dehydrated, embedded in paraffin, and sectioned. Tissue sections were deparaffinized, rehydrated, and incubated for 30 min in 0.3% hydrogen peroxide in methanol and then for 10 min with 1% goat serum in TBS. Then the sections were incubated with rabbit anti-human anti-DNMT1 antibody (Abcam), DNMT3a (Epitomics) and DNMT3b (Imagenex; all at 1:100) at room temperature overnight. After washing three times in TBS, the sections were incubated with biotinylated mouse anti-rabbit IgG (1:5000; Abcam) for 30 min and followed by three 5 min wash in TBS. The final incubation was for 30 min with HRP-avidin D at 37°C. The peroxidase was detected with 0.05% 3,3-diaminobenzidine tetrahydrochloride (DAB). The sections were counterstained with hematoxylin and mounted in neutral gum medium for light microscopy [21]. Positive protein expression was visualized as nuclear localization of granular brown-yellow precipitate. The counts were performed in 3 high power fields of vision under a high magnification (400×) for each section. The percentage of positive cells was calculated as the ratio of positive cells to the total number of cells. The scoring scale for the percentage of positive cells was: 0, less than 1%; 1, 1 - 24%; 2, 25 - 50%; 3, 51 - 75%; 4, more than 75%. The scoring scale for staining intensity was: 0, no color; 1, bright yellow; 2, yellow; 3, brown yellow; 4, brown. The final score was obtained by multiplying the percentage of positive cells by the staining intensity score.

### Statistical analysis

All data were plotted as mean ± standard deviation. Statistical analysis was performed with SPSS 13.0 software. (SPSS Inc., Chicago, IL). Student's t test was used for comparisons. Differences were considered significant when the *P *was less than 0.05.

## Results

### The continuous and low-energy ^125^I seed irradiation-induced cell apoptosis

**Figure 2:**
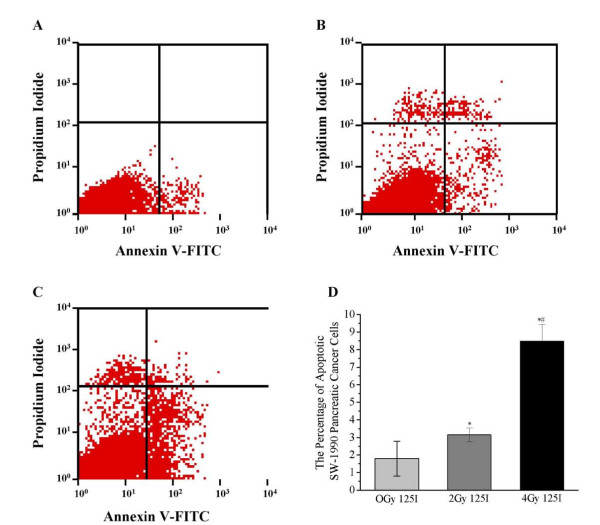
**Apoptosis of ^125^I irradiated SW-1990 cells**. The red region in the lower left quadrant represents apoptosis detected by flow cytometry in the 0 Gy (A), 2 Gy (B), and 4 Gy (C) groups. The quantitation is shown in D. *P < 0.05 compared with the 0 Gy (Control) group. ^#^*P *< 0.05 compared with the 2 Gy group.

The red region in the lower left quadrant and right quadrant represented the survival and apoptosis of cells, respectively. The red region area in lower quadrant in 2 Gy group was slightly bigger than that in 0 Gy group (Figure 2A and 2B). The percentage of apoptotic cells (3.15 ± 0.38%) in 2 Gy group was slightly more than that in 0Gy group (1.78 ± 1.01%) (P < 0.05) (Figure 2D). More importantly, the 4 Gy group exhibited a significantly expanded red area relative to the 2Gy and 0 Gy group (Figure 2A, B and 2C). The percentage of apoptotic cells was substantially more in 4Gy group (8.47 ± 0.96%) than in 2 Gy or 0 groups. (P < 0.01) (Figure 2D). Quantitative measurements of apoptotic cell suggested that apoptosis is an important mechanism of low-energy ^125^I seed irradiation inhibition of SW-1990 cancer cells.

### Expression changes of DNMTs in SW-1990 cells after ^125^I seed irradiation

**Figure 3:**
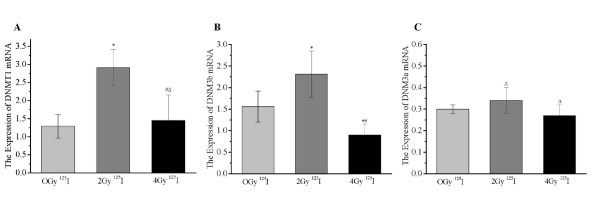
**^125^I irradiation induced expression changes of DNA methyltransferases mRNA in SW-1990 cells**. DNMT1 (A), DNMT3a (B), and DNMT3b (C) mRNA expression in ^125^I irradiated SW-1990 cells was detected as described in the Materials and Methods section. *P < 0.05 compared with the 0 Gy (Control) group. ^#^*P *< 0.05 compared with the 2 Gy group. ^Δ^*P *> 0.05 compared with the 0 Gy group.

Expression of DNMT1 (2.91 ± 0.5) and DNMT3b (2.31 ± 0.54) mRNA in the 2 Gy group was significantly higher than in the 0 Gy group (1.29 ± 0.33 and 1.56 ± 0.36, *P *< 0.05; Figure 3A and 3B). Conversely, the 4 Gy group exhibited a significant decrease in DNMT1 expression (1.45 ± 0.70) and DNMT3b (0.90 ± 0.25) mRNA compared with the 2 Gy group (*P *< 0.05; Figures 3A and 3B). More importantly, DNMT3b expression was lower in the 4 Gy group (0.90 ± 0.25) than in the 0 Gy group (1.56 ± 0.36, *P *< 0.05; Figure 3B). Moreover, DNMT3a mRNA expression did not differ among the three groups (Figure 3C). These data suggest that ^125^I seed irradiation significantly affects the expression of DNMT1 and DNMT3a mRNA.

**Figure 4:**
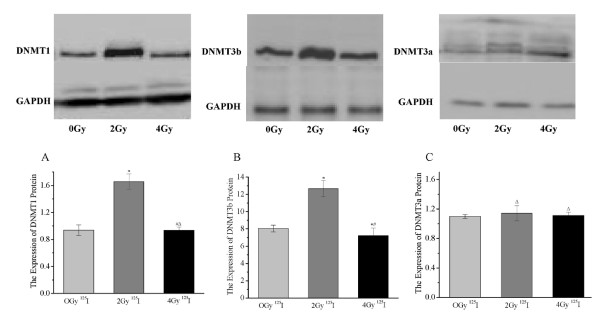
**^125^I irradiation altered DNMTs protein expression in SW-1990 cells**. Representative western blots of DNMT proteins are showed in the upper panel. DNMT1 (A), DNMT3a (B), and DNMT3b (C) protein expression in ^125^I irradiated SW-1990 cells was detected as described in the Materials and Methods section. *P < 0.05 compared with the 0 Gy (Control) group. ^#^*P *< 0.05 compared with the 2 Gy group. ^Δ^*P *> 0.05 compared with the 0 Gy group.

Representative western blots for DNMTs are shown in the upper panel of Figure 4. The ratios of DNMTs to GAPDH density were calculated to determine protein expression levels. DNMT1 (1.65 ± 0.11) and DNMT3b (12.65 ± 0.94) protein expression were dramatically higher in the 2 Gy group than in the 0 Gy group (0.93 ± 0.07 vs. 8.04 ± 0.39, *P *< 0.05; Figures 4A and 4B). DNMT1 (0.93 ± 0.04) and DNMT3b (7.32 ± 0.85) protein expression decreased further in the 4 Gy group compared with the 2 Gy group (*P *< 0.01; Figures 4A and 4B). More importantly, the 4 Gy group (7.32 ± 0.85) exhibited decreased DNMT3b protein expression relative to the 0 Gy group (8.04 ± 0.39, *P *< 0.05; Figure 4B). However, there were no significantly statistical differences in DNMT3a protein expression among the three groups. These data suggest that ^125^I irradiation significantly affects DNMT1 and DNMT3b protein expression.

### The number of apoptotic cells in pancreatic cancer after ^125^I seed implantation

**Figure 5:**
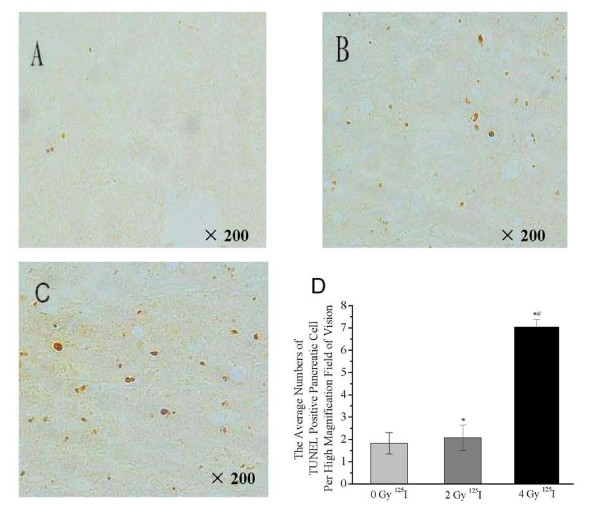
**^125^I irradiation induced apoptosis in pancreatic cancer**. The dark brown or brownish yellow spots represented the apoptotic cells detected by TUNEL staining in the 0 Gy (A), 2 Gy (B), and 4 Gy (C) groups. The average number of apoptotic cells per 200 objective fields were plotted (D). *P < 0.05 compared with the 0 Gy (Control) group. ^#^*P *< 0.05 compared with the 2 Gy group.

The TUNEL-positive apoptotic cells were dark brown or brownish yellow in color. Representative TUNEL stains obtained from the 0 Gy, 2 Gy and 4 Gy groups are showed in Figures 5A, B, and 5C, respectively. The average number of apoptotic cells increased slightly in the 2 Gy group (2.07 ± 0.57) compared to the 0 Gy group (1.83 ± 0.48, *P *< 0.05; Figure 5D). The average number of apoptotic cells in the 4Gy group (7.04 ± 0.34) was significantly higher than in the 2 Gy or 0 Gy group (*P *< 0.01; Figure 5D). These data suggest that the ^125^I seed implantation induced significant apoptosis in pancreatic cancer cells.

### Immunohistochemistrical stains for DNMTs in pancreatic cancer after ^125^I seed implantation

**Figure 6:**
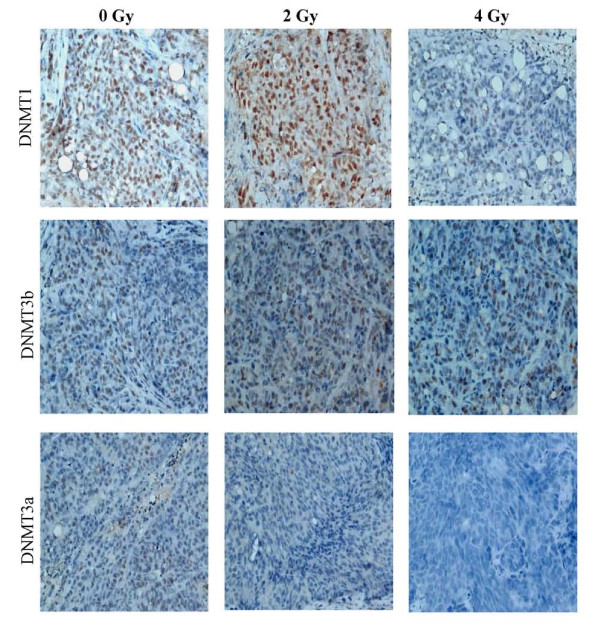
**Immunohistochemical staining for DNMTs in ^125^I seed implanted pancreatic cancer**. Representative staining sections for DNMT1 (upper), DNMT3b (middle) and DNMT3a (lower) were prepared as described in the Materials and Methods section. The brownish yellow spots represent positive staining. Scale bars represent 500 μm.

DNMT1, DNMT3b and DNMT3a protein expression was detected as brownish yellow spots by immunohistochemical staining (upper, middle and lower panel of Figure 6, respectively). The brownish yellow staining for DNMT1 and DNMT3a were more obvious in the 2 Gy group than in the 0 Gy group. However, DNMT1 and DNMT3b staining was significantly weaker in the 4 Gy group compared with the 2 Gy group. More importantly, the brownish yellow for DNMT1 and DNMT3b staining was moderately reduced in the 4 Gy group compared with the 0 Gy group. There were no significant differences in DNMT3a staining observed among the three groups. These data suggest that ^125^I seed implantation prominently altered the expression of DNMT1 and DNMT3b, but not DNMT3a, in pancreatic cancer.

Table 1 showed the quantitation of DNMTs protein positive expression 28 d after ^125^I seed implantation. DNMT1 (9.11 ± 3.64) and DNMT3b (7.27 ± 3.76) protein expression scoring in the 2 Gy group were dramatically higher than in the 0 Gy group (6.72 ± 2.63 and 6.72 ± 2.63, *P *< 0.05). However, in the 4 Gy group, there was a significant decrease in DNMT1 (6.50 ± 2.85) and DNMT3b (4.66 ± 2.17) protein expression compared with 2 Gy group (*P *< 0.01). More importantly, the 4 Gy group (3.11 ± 2.42) exhibited a statistically decreased expression scoring of DNMT3b protein relative to the 0 Gy group (4.72 ± 2.16, *P *< 0.05). Moreover, no significantly statistical differences were observed in DNMT3a protein expression among the three groups. Therefore, the expression changes in DNMTs protein in an animal model was in agreement with those observed in cultured cells subjected to similar ^125^I irradiation.

**Table 1:** The positive expression scoring of DNMTs protein in ^125^I pancreatic cancers

	DNMT1	DNMT3b	DNMT3a
**Control Group (0Gy**)	6.72 ± 2.63	4.72 ± 2.16	2.61 ± 1.24
**2Gy Group**	9.11 ± 3.64*	7.27 ± 3.76*	3.22 ± 1.30^Δ^
**4Gy Group**	6.50 ± 2.85^#Δ^	3.11 ± 2.42*^#^	3.06 ± 2.13^Δ^

DNMT, DNA methyltransferases.

*P < 0.05 compared with 0 Gy (Control) group. ^#^*P < 0.05 *compared with 2 Gy group. ^Δ^*P > 0.05 *compared with 0 Gy group.

### Histopathology of in pancreatic cancer after ^125^I seed implantation

**Figure 7:**
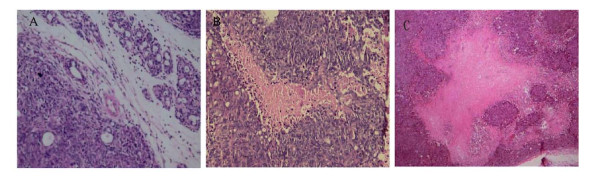
**Pathology of ^125^I implanted pancreatic cancer**. Representative HE stained sections from the 0 Gy (A), 2 Gy (B), and 4 Gy (C) groups 28 d after ^125^I seed implantation were prepared as described in the Materials and Methods section.

Representative HE sections were obtained from the 0 Gy (Figure 7A), 2 Gy (Figure 7B), and 4 Gy (Figure 7C) groups 28 d after ^125^I seed implantation. In the 0 Gy group, there was no significant necrotic or damaged regions. The cancer cells were densely arranged in a disorderly fashion, with large, darkly stained nuclei with obvious fission. In the 2 Gy and 4 Gy groups, a large area of coagulative necrosis was observed around the ^125^I seed; also the surviving cells adjacent to the necrotic region were loosely arranged, with nuclear condensation and decreased eosinophilia of the cytoplasm. The cancer cells in the submucosal layer were tightly packed with nuclear condensation of discrete cells. More importantly, the necrosis and growth inhibition in cancer cells were more obvious in 4Gy group than in 2 Gy group. These suggestion that ^125^I seed implantation caused the necrosis and growth inhibition of cancer cells and enlargement of irradiation dose could enhance the beneficial effect.

### Tumor volume of pancreatic cancer at 0 and 28 days after ^125^I seed implantation

**Figure 8:**
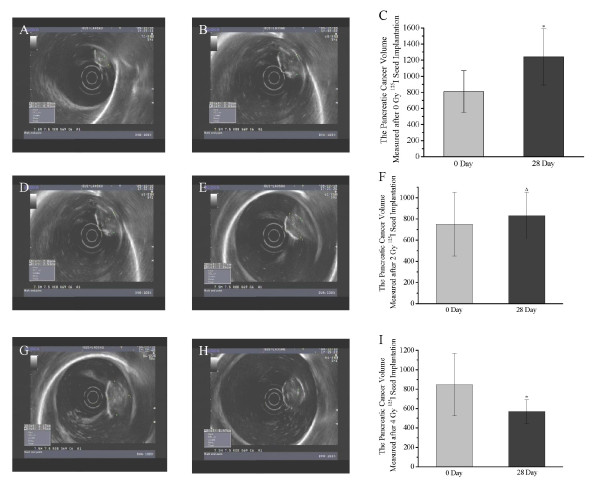
**Tumor volume 0 and 28 d after ^125^I seed implantation**. The upper, middle, and lower panels show representative ultrasound images from 0 Gy (upper), 2 Gy (middle), and 4 Gy (lower) groups 0 and 28 d post ^125^I seed implantation. *P < 0.05 compared with 0 d post-implantation; ^Δ^*P *> 0.05 compared with 0 d post-implantation.

Representative ultrasonic images from 0 and 28 d after implantation of ^125^I seed in the 0 Gy, 2 Gy, and 4 Gy groups are shown in Figure 8. Quantitative measurements of tumor volume in the 0 Gy, 2 Gy, and 4 Gy groups are shown in Figure 8C, F, and 8I, respectively. In the 0 Gy group, pancreatic cancer proliferated rapidly from 0 d to 28 d after implantation (Figures 8A and 8B). The tumor volume (1240 ± 351 v/mm^3^) at 28 d was significantly larger than at 0 d (809 ± 261, *P *< 0.01; Figure 8C). No significant alteration in tumor volume was observed between 0 d and 28 d in the 2 Gy group (Figures 8D and 8E). There was no statistical difference in the tumor volume between 0 d and 28 d in the 2 Gy group (750 ± 300 vs. 830 ± 221, *P *> 0.05; Figure 8F). More importantly, the 4 Gy group demonstrated that the treatment effectively eliminated the tumor (Figures 8D and 8E). The tumor volume decreased dramatically, from 845 ± 332 at 0 d to 569 ± 121 at 28 d (*P *< 0.01; Figure 8I). These results suggest that ^125^I seed implantation inhibits tumor growth and reduces tumor volume, with 4 Gy being more effective than 2 Gy.

## Discussion

Epigenetic changes in cells are closely linked to tumor occurrence, progression and metastases. DNA methylation is a crucially important epigenetic alteration by which the tumor suppressor gene expression and cell cycle regulation may be substantially altered. Three different DNMTs, specifically DNMT1, DNMT3a and DNMT3b, have critical roles in establishing and maintaining DNA methylation. Many chemotherapeutic agents exert their antitumor effects by inducing apoptosis in cancer cells. The purpose of this study is to investigate whether ^125^I seed irradiation significantly influences the expression of DNA methyltransferases, promote the cell apoptosis and inhibit the pancreatic cancer growth.

SW-1990 pancreatic cancer cells were cultured *ex vivo *and implanted into the pancreas to create the animal model. The ^125^I seed irradiation induced apoptosis in SW-1990 cells. Likewise, large numbers of apoptotic cells were present in pancreatic cancer receiving ^125^I seeds implantation. Irradiation-induced apoptosis became more obvious when the radiation dose increased from 2 Gy to 4 Gy. DNMT1 and DNMT3b mRNA and protein expression was increased substantially in 2 Gy ^125^I irradiated SW-1990 cells, whereas ^125^I irradiation with 4 Gy inhibited DNMT3b mRNA and protein expression. The expression of DNMT3a mRNA did not change regardless of the ^125^I irradiation dose. The similar DNMT expression patterns were confirmed by immunohistochemical staining in ^125^I seed implanted pancreatic cancer. Most importantly, the 2 Gy ^125^I seed implantation limited the growth of the pancreatic tumor, while 4 Gy ^125^I seed implantation substantially decreased pancreatic tumor volume.

Our results demonstrated that apoptosis may have an important role in the therapeutic effects when pancreatic cancer is exposed to continuous low-energy ^125^I irradiation. The apoptosis in the 4 Gy group was more obvious than in the 2 Gy group, which is in agreement with the fact that cancer treatment is more effective at 4 Gy than at 2 Gy. Similar irradiation-induced apoptosis patterns were also observed in the other cancer cell lines [22]. The ^125^I irradiation induced apoptosis was the primary mechanism of CL187 colonic cancer cell-killing under low dose treatment [22]. Ionizing radiation can generate the reactive oxygen species (ROS), which induce apoptosis [23]. The ROS damages critical cellular components such as DNA, proteins, and lipids, eventually causing cellular apoptosis [24]. Therefore, the ^125^I irradiation-induced apoptosis is a key mechanism underlying the therapeutic effect of ^125^I seed implantation in pancreatic cancer.

Our results demonstrated that altered DNA methylation patterns might have a pivotal role in tumor inhibition resulting from consecutive low-energy irradiation. The 2 Gy irradiation caused a significant increase in DNMTs expression, whereas 4 Gy irradiation was associated with decreased DNMTs expression. However, a substantial reduction in tumor volume was only observed in 4 Gy irradiation group rather than in 2 Gy group at 28 d after ^125^I seed implantation. There are a strong and positive correlation between DNA methylation and expression of DNMTs, because DNMTs maintain DNA methylation patterns [25]. Therefore, it is reasonable to speculate that DNA hypomethylation significantly inhibits cancer cell proliferation or impairs cell survival potentially to an even greater extent than DNA hypermethylation. X- and γ-radiation induce DNA hypomethylation paralleled by decreased DNMTs expression in somatic cells [25-28]. Actually, low-dose irradiation (2Gy) predominantly resulted in reversible DNA damage, which was associated with DNA repair. The DNMTs are the key enzyme for DNA repair. As a result, the increase in reactive DNMTs expression reflects active DNA repair. Thus, ^125^I irradiation-induced DNA hypomethylation could be the key mechanism by which ^125^I seed implantation lead to tumor growth inhibition.

Aberrant *de novo *DNA methylation is commonly associated with cancer, and DNA methylation in mammalian cells largely occurs on cytosine residues at CpG dinucleotides in genomic DNA. The *de novo *methylation of CpG islands in promoter regions of tumor suppressor genes can result in silencing [29]. Thus, DNA hypermethylation might lead to cancer generation and progression [29]. The irradiation-induced DNA demethylation, as the result of decreased DNMTs expression, can reactivate the tumor suppressor gene and inhibit tumor growth. The inhibitory effect of DNA demethylation on cancer was also demonstrated by the demethylating agent 5-aza-cytidine (AZA) and zebularine. Incorporation of a demethylating agent (like a cytidine analog) into DNA during replication inhibited DNMTs enzyme activity and demethylated the tumor suppressor genes, eventually leading to tumor growth inhibition [30,31]. AZA demethylates the P16 and pMLHI gene promoters and reactivates these previously silenced tumor suppressor genes [30,32]. Zebularine administration depleted DNMT1 and the demethylation of the P16 and RASSFIA gene promoters [33,34]. Activation of the tumor suppressor genes RASSF1A and P16 inhibited cell proliferation by inhibiting accumulation of cyclin D, which arrests cell cycle progression at the G1/S phase transition [35]. G1 includes a restriction point beyond which the cell is committed to undergo division independent of growth regulatory signals. As a result, the mechanisms underlying the inhibitory effect of DNA hypomethylation on tumors could be related to reactivating tumor suppressor genes and negative regulation of cell cycle progression.

In conclusion, our study provides important insight into the mechanism by which ^125^I seed irradiation affects pancreatic cancer. ^125^I seed implantation effectively inhibited tumor growth and reduced tumor volume, especially at 4 Gy. ^125^I irradiation-induced apoptosis and DNA hypomethylation are two key mechanisms underlying the therapeutic effect of low-energy ^125^I seed implantation.

## Competing interests

The authors declare that they have no competing interests.

## Authors' contributions

J.M. carried out the molecular genetic studies, participated in the sequence alignment and drafted the manuscript. P.S., Y.L.and Z.L. participated in preparation of animal model. H. W. was responsible for cell culture. X.P. and L.W. particiated in the immunohistochemistry. Y.G., J.G., and Z.L. participated in the design of the study and performed the statistical analysis. Z.J. conceived of the study, and participated in its design. All authors read and approved the final manuscript.
